# Re: Racial and Sociodemographic Disparities in Hepatitis C Treatment at an Urban Academic Medical Center, 2018-2023

**DOI:** 10.1093/ofid/ofaf720

**Published:** 2025-12-03

**Authors:** Heather Lopez, Jeffrey D Klausner, Chrysovalantis Stafylis

**Affiliations:** Department ofPopulation and Public Health Sciences, University of Southern California, Los Angeles, California, USA; Department ofPopulation and Public Health Sciences, University of Southern California, Los Angeles, California, USA; Department ofPopulation and Public Health Sciences, University of Southern California, Los Angeles, California, USA

To the editor—We read with great interest the study by Elnaiem et al. describing that only 26.5% of people with hepatitis C infection were treated in an urban academic medical center and that treatment rates varied across insurance type: Medicare, 55%; private commercial, 48%; Medicaid, 32%; and no insurance, 21% [1]. Those with Medicaid and no insurance experienced the lowest treatment rates. The results of the study were surprising given the urban setting and that the state of Massachusetts publicly maintained progressive treatment policies on hepatitis C virus (HCV) treatment medication by streamlining prior authorization and had few or no restrictions based on fibrosis stage, sobriety, or prescriber throughout the study period.

We confirmed these findings in another large urban area, Los Angeles County. Our team operates Project HCV Connect, a university-based volunteer linkage-to-cure case management program using the Los Angeles County hepatitis C virus surveillance registry [2]. Findings from our project show that 69% are untreated, as shown in the Table 1. There were somewhat higher frequencies of lack of treatment in those with either no or public insurance versus those with commercial insurance, although the lack of treatment of those with commercial insurance was still high at 66%, as indicated in the Table 1.

**Table 1.** Treatment Status by Insurance Among Participants of Project HCV Connect With a Reported Positive hepatitis C virus RNA Test Result Between 2020 and 2025 (N = 1119)

Insurance Type	N	Untreated (%)
Commercial	267	176 (65.9)
Medicare	206	156 (75.7)
Medicaid (Medi-Cal)	528	372 (70.5)
Other public-sponsored insurance	7	5 (71.4)
No insurance	45	38 (84.4)
Decline to answer	66	21 (31.8)
Total	1119	768 (68.6)

California's Medicaid program removed prior authorization restrictions for hepatitis C treatment medications in 2024 [3]. This population still accounts for the largest absolute number of untreated patients in our sample. These findings, along with those of Elnaiem et al., indicate that although removing prior authorization restrictions are a necessary and critical step toward achieving hepatitis C elimination, the Medicaid population is still disproportionately disadvantaged. Elnaiem et al.'s findings identified homelessness and substance use disorder as significant predictors of low hepatitis C virus treatment rates [1]. In California alone, the U.S. state with the highest number of people experiencing housing instability, >70% of unsheltered persons were enrolled in the state's Medicaid program, Medi-Cal [4]. Additionally, almost half of adults in California with a substance use disorder are enrolled in Medi-Cal [5]. It is crucial to address these issues within the Medi-Cal population to see the full benefit of prior authorization removal.

However, anyone insured outside of Medi-Cal may still be subject to prior authorization barriers, as these restrictions are not removed the way they are in Medi-Cal. In a qualitative study of provider perspectives on barriers to hepatitis C virus treatment delivery, hepatitis C virus treatment providers agreed that prior authorization is the greatest obstacle causing a delay in treatment initiation [6]. Prior authorization restrictions in health insurance can also cause loss to follow-up, act as a resource burden on an already overwhelmed healthcare system, and thereby contribute to the large number of untreated people. At the policy level, until prior authorization restrictions for hepatitis C treatment are removed across all insurance types, equitable hepatitis C virus elimination cannot be achieved.

In the United States, most private and other public insurance plans impose prior authorization requirements for hepatitis C virus treatment, causing delays and drop offs in treatment initiation [6]. We agree with the authors that health insurance companies need to prioritize streamlining and preferably eliminate the prior authorization processes, as 29 U.S. states have already done in Medicaid programs [3]. Untreated cases with hepatitis C virus will inevitably cause ongoing transmission, preventable liver disease, and premature deaths. Elnaiem et al.'s article showed that untreated people had a higher frequency of inpatient and emergency department visits than those who had already been treated [1]. Additionally, in the United States, the total direct economic burden of HCV-related liver disease is more than $10 billion per year [7]. It is in the best interest of healthcare insurance companies to remove prior authorization to reduce the long-term cost by facilitating treatment access now, preventing future complications, medical visits, and continued transmission. With prior authorization removed across all insurance programs, we can take another step forward to eliminate hepatitis C virus infection in the United States.

## References

[1] Elnaiem AD, Chukka AB, So-Armah CM, et al Racial and sociodemographic disparities in hepatitis C treatment at an urban academic medical center, 2018–2023. Open Forum Infect Dis 2025; 12:ofaf312. 40519633 10.1093/ofid/ofaf312 PMC12164289

[2] Los Angeles County Department of Public Health . Viral hepatitis. Available at: http://ph.lacounty.gov/acd/diseases/hepatitis/home.htm Accessed 2 October 2025.

[3] Center for Health Law and Policy Innovation, & National Viral Hepatitis Roundtable . Hepatitis C: State of Medicaid access. 2024. Available at: https://stateofhepc.org. Accessed 2 October 2025.

[4] Kushel M, Moore T. Toward a new understanding: the California statewide study of people experiencing homelessness. University of California, San Francisco. 2023. Available at: https://homelessness.ucsf.edu/sites/default/files/2023-06/CASPEH_Report_62023.pdf. Accessed 29 October 2025.

[5] California Behavioral Health Director's Association . 2015. Expanding California's capacity to treat individuals with substance use disorders (CBHDA Governing Board Policy Brief). Available at: https://healthlaw.org/resource/substance-use-disorders-in-medi-cal-an-overview/. Accessed 29 October 2025.

[6] Javanbakht M, Archer R, Klausner J. Will prior health insurance authorization for medications continue to hinder hepatitis C treatment delivery in the United States? Perspectives from hepatitis C treatment providers in a large urban healthcare system. PLoS One 2020; 15:e0241615. 33147293 10.1371/journal.pone.0241615 PMC7641373

[7] Stepanova M, Younossi ZM. Economic burden of hepatitis C infection. Clin Liver Dis 2017; 21:579–94. 28689595 10.1016/j.cld.2017.03.012

